# Neural Representation of Parental Monitoring and Links to Adolescent Risk Taking

**DOI:** 10.3389/fnins.2019.01286

**Published:** 2019-12-03

**Authors:** Tae-Ho Lee, Yang Qu, Eva H. Telzer

**Affiliations:** ^1^Department of Psychology, Virginia Tech, Blacksburg, VA, United States; ^2^School of Education and Social Policy, Northwestern University, Evanston, IL, United States; ^3^Department of Psychology and Neuroscience, The University of North Carolina at Chapel Hill, Chapel Hill, NC, United States

**Keywords:** adolescence, risk taking, parental monitoring, fMRI, representational similarity analysis

## Abstract

Decades of developmental research have demonstrated the positive role of parental monitoring during adolescence, a time during which youth seek exploration and show heightened risk taking. The present study employed a novel neural pattern similarity approach to identify neural patterns underpinning parental monitoring, with attention to implications for adolescent risk taking. Mothers (*N* = 23) underwent an fMRI scan during which they completed a risk-taking task and viewed the risk-taking behavior of their adolescent child. Using a representational similarity analysis, we examined the neural pattern similarity between mothers’ anticipation of their child’s risk taking and their own decisions. Higher parental monitoring was reflected in greater similarity between neural pattern of anticipating their adolescents’ risk taking and experiencing their own safe outcomes. Moreover, greater neural pattern similarity between mothers’ anticipation and their own safe outcomes was associated with lower risk-taking propensity in adolescents. Taken together, the present study provides preliminary evidence for the neural patterns underpinning parental monitoring, highlighting the importance of incorporating parents’ brain as a window to understand parenting practices and adolescent risk taking.

## Introduction

Adolescence is a time of individuation and exploration, during which youth are more likely to engage in problem behaviors, such as delinquency and substance use (Arnett, 1992; Karriker-Jaffe et al., 2008; Martino et al., 2008). As active socializers, parents play an important role during this phase of development (for reviews, see Collins and Steinberg, 2006; Smetana et al., 2006). One key parenting practice is parental monitoring – the effort that parents take to obtain information concerning their adolescents’ activities, whereabouts, and associations (Dishion and McMahon, 1998; Stattin and Kerr, 2000; Fletcher et al., 2004). Decades of research in developmental psychology has documented the positive role of parental monitoring on adolescents’ adjustment. For example, empirical evidence based on both cross-sectional and longitudinal studies suggests that higher levels of parental monitoring are linked to better academic achievement, lower delinquency, and less health-compromising risk-taking behaviors, such as drinking, smoking, unprotected sexual behavior, drug use, and antisocial behavior (Steinberg et al., 1994; Jacobson and Crockett, 2000; Li et al., 2000a; DiClemente et al., 2001; Mann et al., 2015). The current research aimed to identify the neurobiological representation of parental monitoring by employing an innovative neural pattern similarity approach to compare parents’ anticipation of their adolescent child’s risk taking and their own risk-taking decisions, with attention to its implications for adolescents’ risk taking.

Prior neuroimaging studies have investigated the role of parents on adolescent risk taking by focusing on the adolescent brain. For example, the presence of mothers leads to momentary changes in adolescent neural activation in the ventral striatum during risk taking and promotes safe decisions (Telzer et al., 2015). Moreover, the characteristics of parents and parent-child relationships, such as parental depression and parent-child conflict, also relate to adolescent brain development, including longitudinal changes in ventral striatum and lateral prefrontal cortex activation, which is related to changes in adolescent risk taking over time (Qu et al., 2015, 2016; McCormick et al., 2016). Although this approach provides valuable insights into the processes through which parents or parental practices influence adolescent brain, it remains unknown how parents’ neural processes that underlie parenting practices or behavior relate to adolescent risk taking.

Neuroimaging research that examines neural representation of parenting practices or beliefs is scarce. For example, a recent study suggests that when parents make judgments about their parenting competence, they recruit greater activity in the medial prefrontal cortex, a neural region involved in general trait self-evaluation (Noll et al., 2018). Moreover, prior research has explored the neural processes underlying parents’ response to their children’s distress. For example, parental empathic experiences and neural engagement of the social brain network (e.g., temporo-parietal junction) are more intensified when parents see their child’s pain (Leibenluft et al., 2004; Goubert et al., 2008) regardless of the parent-child relationship quality with their child (Lee et al., 2017). Although such emerging evidence suggests the importance of investigating parents’ neural processing as a window to understand parents’ beliefs and practices, it is unclear whether neural processes of parenting practices are linked to children’s adjustment. Advances in this endeavor will strengthen the value of incorporating parents’ brain into the understanding children’s adjustment.

The current study aimed to examine neural representation of parental monitoring using a representational similarity approach. To this end, parent-adolescent dyads participated in a series of tasks. Adolescents first completed a widely used task that mimics adolescent risk taking (the “Stoplight Task”; Chein et al., 2011). During the fMRI scan, mothers completed two runs of the Stoplight task, the order of which was counterbalanced. In one run, mothers played the Stoplight Task (i.e., ‘Driving’ run), during which they could make safe (i.e., stop at yellow light) or risky (i.e., go through yellow light and risk crashing into oncoming car) decisions, which measures mothers’ neural activity when they are actively performing the Stoplight task. In a another run, the ‘Observation’ run, mothers were presented a video recording of their child’s Stoplight performance and asked to view it passively. Given that mothers only know their child’s decisions after seeing the outcome, we were particularly interested in the time period before the outcome appeared, because it can reflect mothers’ anticipation of their child’s decisions. To better characterize mothers’ anticipation, we employed a representational similarity approach, which allows us to compare the neural patterns during mothers’ anticipation of their child’s decisions and mothers’ own decisions. Different from univariate approaches that only focus on differences in neural activation across conditions, the representational similarity approach can better quantify how similar these patterns are across conditions. For example, if the neural patterns during anticipation estimated from the ‘Observation’ run are similar to neural patterns when mothers experienced a ‘Safe’ outcome (i.e., following their own “stop” decision) during the ‘Driving’ run, which would suggest that mothers’ neural representation of anticipating their child’s behavior is more similar to their own safe decisions. If the neural patterns during anticipation estimated from the ‘Observation’ run are similar to neural patterns when mothers experienced a ‘Risky’ outcome (i.e., following their own “go” decision) during the ‘Driving’ run, it would suggest that mothers’ neural representation of anticipating their child’s behavior is more similar to their own risky decisions. Given that parental monitoring represents parents’ effort to anticipate and know their children’s behavior, we first conducted exploratory analyses and examined whether the pattern similarity between mother’s anticipation and their decisions can serve as neural representation of parental monitoring. We hypothesized that parents’ greater effort to know their children’s behavior (i.e., greater parental monitoring) may be linked with greater neural similarity between mother’s anticipation and their own safe decisions. We further sought to investigate how the neural representation of parental monitoring is associated with adolescents’ risk-taking propensity. We hypothesized that the greater neural similarity between mother’s anticipation of their child’s decisions and their own safe decisions, serving as a neural index of greater parental monitoring, would be related to lower risk-taking propensity in adolescents.

## Materials and Methods

### Participants

Twenty-three mothers (*M*_*age*_ = 44.61 years, SD = 6.81, range: 32–64) and their adolescent child (*M*_*age*_ = 13.83 years, SD = 0.49, range: 13–14) participated in this study. Mothers and children underwent fMRI scans on the same day, but children’s scan happened in advance of their mothers’ scan (see Stoplight task and procedure)^1^. All mothers provided written informed consent and adolescent participants provided written assent, approved by the Institutional Review Board of the University of Illinois, Urbana-Champaign.

### Measures

#### Parental Monitoring

To measure how actively mothers monitor their child’s behavior in their daily life, mothers completed the Parental Monitoring Scale (Stattin and Kerr, 2000), which consisted of 10 items on a 5-point Likert scale from 1 (never) to 5 (very often). Sample items included “Before your child went out, you asked him/her where he/she was going,” “You told your child to call you if he/she was going to be late getting home,” and “You asked your child who he/she was going to be with before he/she went out” (reliability α = 0.844). The ten items were averaged, and higher scores indicate greater monitoring of their child’s daily behavior.

#### Adolescents’ Risk-Taking Propensity

To measure adolescent risk taking, youth completed a modified version of the Domain-Specific Risk-Taking (DOSPERT) scale, a well-vaidated measure of the likelihood of engaging in risky events (Weber et al., 2002; Figner and Weber, 2011; Barkley-Levenson et al., 2013). Adolescents reported on how likely they would engage in a variety of risky behaviors (39 items) using a 7-point Likert scale from 1 (extremely unlikely) to 7 (extremely likely). Sample items included “Drinking at a party,” “Stealing something from the newsstand,” and “Engaging in unprotected sex” (reliability α = 0.903). The items were averaged, and higher scores indicate greater likelihood of engaging in risk taking in daily life.

### Stoplight Task and Procedure

**FIGURE 1:**
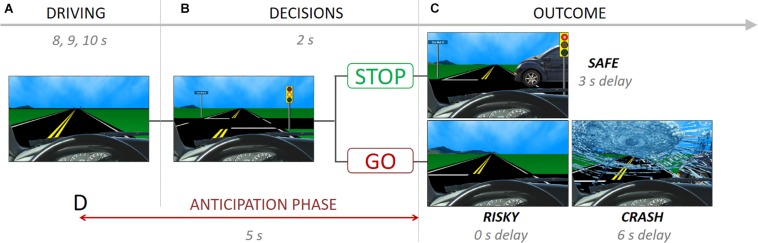
The stoplight task showing **(A)** driving phase, **(B)** decision phase and possible choices, **(C)** outcome phase and possible consequences, and **(D)** anticipation phase.

The Stoplight task measures risk taking at the behavioral and neural level (Gardner and Steinberg, 2005; Chein et al., 2011; Telzer et al., 2015; Guassi Moreira and Telzer, 2018). During the task, participants take the perspective of a person driving a car and encounter 26 yellow stoplight intersections, and are instructed to finish the driving course as fast as possible (Figure 1A). Participants have to decide whether to ‘Stop’ or ‘Go’ at each yellow stoplight (Figure 1B). A ‘go’ decision is the fastest option to pass through the intersection (outcome: ‘Risky’), but participants run the risk of crashing, resulting in a 6-s delay (outcome: ‘Crash’; Figure 1C). If participants choose to ‘Stop,’ they do no risk crashing, but it results in a 3-s delay at the intersection, leading the task to be longer (outcome: ‘Safe’). Thus, ‘Go’ decisions reflect risky decision-making whereas ‘stop’ decisions represent safe decision-making. Each intersection was spaced by 8, 9, or 10s inter-trial intervals. There was a 30% probability of crashing across the 26 intersections, which was not explicitly disclosed to participants so as not to influence their decision making, but they were told that there was a chance of crashing at any intersection depending on the decision they made. The schedule of a car-crossing intersections was pseudo-randomly assigned.

During the fMRI scan, mothers completed two runs of the Stoplight task. In one run, mothers played the Stoplight Task (i.e., ‘Driving’ run), which measures mothers’ neural activity when they are actively performing the Stoplight task. In a second run, the ‘Observation’ run, mothers were presented a video recording of their child’s Stoplight performance and asked to view it passively. For the observation run, each mother’s child played the Stoplight task in the scanner in advance, and we recorded the screen of their behavior using Screencast-O-Matic software^2^.

### fMRI Data Acquisition and Analysis

#### Image Acquisition and Preprocessing

Imaging data were collected using a 3T-Siemens Trio MRI scanner with a 16-channel matrix coil. High-resolution structural images (T1-MPRAGE) were acquired (repetition time or TR = 1.9 s; echo time or TE = 2.3 ms; matrix size = 256 × 256; field of view or FOV = 230 mm; flip angle or FA = 90°; voxel size = 0.9 × 0.45 × 0.45 mm). T2^∗^-weighted echo-planar images (EPI) were acquired during the Stoplight task (38 slices with 0.3 mm inter-slice gap; TR = 2 s; TE = 25 ms; matrix = 92 × 92; FOV = 230 mm; FA = 90°; voxel size = 2.5 × 2.5 mm; slice thickness = 3 mm). Preprocessing for the pattern analysis was carried out using FEAT (fMRI Expert Analysis Tool) Version 6.00, part of FSL (FMRIB’s Software Library; Smith et al., 2004). The following pre-statistics processing was applied; motion correction using MCFLIRT (Jenkinson et al., 2002; mean FD = 0.098 mm, SD = 0.042 mm, range: 0.041–0.246 mm); non-brain removal using BET (Smith, 2002); grand-mean intensity normalization of the entire 4D dataset by a single multiplicative factor; 128-s highpass filter.

#### ROI Selection

**FIGURE 2:**
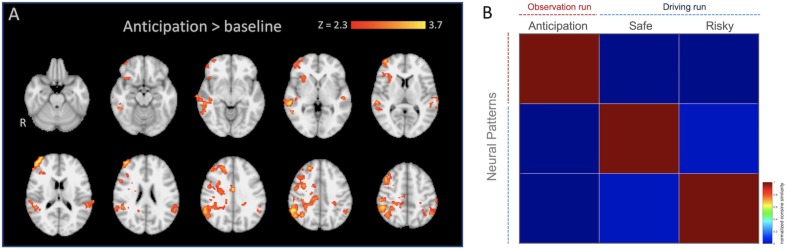
**(A)** Anticipation network ROI mask used for the pattern similarity (radiological). **(B)** Averaged similarity matrix as a function of neural patterns across mothers. Note that the averaged similarity matrix is included in the figure, it should not be interpreted inferentially.

We created regions of interest (ROIs) by performing an additional standard two-stage mixed effects whole-brain univariate analysis using the observation run in which the anticipation regressor (see below) was modeled individually with temporal derivate regressor and nuisance regressors, and individual level anticipation contrasts were inputted into group level analysis (FLAME 1 + 2; *Z* > 2.3; one-tailed *P* = 0.05). We selected all voxels from [anticipation > baseline] contrast (*k*, a number of voxels = 9363), and used them as our anticipation network ROI mask for the pattern extraction. For this analysis, we applied 6-mm smoothing, ICA denoising using an automated signal classification toolbox (Tohka et al., 2008), and spatial normalization for 2-mm MNI template using ANTs (Avants et al., 2011) for individual data. As shown in Table 1 and Figure 2A, mothers recruited greater activity in regions such as the anterior cingulate cortex (ACC), orbital frontal cortex (OFC), temporoparietal junction (TPJ), and insula during anticipation compared with baseline^3^.

**TABLE 1:** Brain regions within significant clusters on the observation run between anticipation phase and baseline.

					**MNI coordinates**		
	**H**	**Z**	***k***	***BA***	***x***	***y***	***z***
**Anticipation > baseline**							
Supramarginal Gyrus posterior	R	4.33	184	40	50	−42	52
Temporoparietal Junction (TPJ)	R	3.29	343	39	58	−52	30
Frontal Pole	R	4.12	788	–	42	56	14
Superior Temporal Gyrus posterior	R	3.94	97	–	60	−32	2
Anterior Cingulate Cortex (ACC)		3.80	97	24	2	−4	30
Middle Temporal Gyrus posterior	R	3.78	167	21	60	−34	−2
Supramarginal Gyrus anterior	R	3.48	41	40	56	−28	50
Lateral Occipital Cortex superior	R	3.46	166	39	54	−60	40
Middle Frontal Gyrus	R	3.45	212	8	46	22	44
Middle Temporal Gyrus	R	3.38	66	37	58	−50	−4
Planum Temporale	L	3.19	37	22	−58	−36	16
Posterior Cingulate Cortex (PCC)		3.18	59	31	12	−26	42
Postcentral Gyrus	R	3.09	35	–	52	−24	46
Supramarginal Gyrus posterior	L	3.06	64	39	−64	−48	22
Temporoparietal Junction (TPJ)	L	3.17	23	39	−54	−52	34
Orbital Frontal Cortex (OFC)	R	3.00	48	47	42	22	−14
Inferior Frontal Gyrus (vlPFC)	R	2.99	20	38	54	14	24
Superior Parietal Lobule	R	2.99	55	7	34	−44	50
Precentral Gyrus	R	2.97	64	6	54	−4	42

*The reported region labels are from the Harvard-Oxford atlas at 50% probability locations with more than 20 voxels; H, hemisphere (R, right; L, left); Z, *z*-value; k, number of contiguous voxels; BA, Broadman Area.*

#### Anticipation ROI

We selected and used all functional voxels activated during the anticipation phase for the analysis, instead of running the analysis for each region separately. We ran the similarity analysis using this global network level ROI mask (i.e., including all possible regions involved in the anticipatory processing). Anticipation does not recruit a single region but is a complex mental process recruiting different brain regions simultaneously to mentalize about children’s behavior (e.g., TPJ, PCC), integrate incoming sensory and bottom-up emotional inputs (e.g., amygdala, OFC, LOC), and regulate induced anxiety (e.g., PFC regions). In this context, we did not test how and what single brain region is involved in the anticipation, but how the network of regions involved in anticipatory processing in general represent mothers’ parental monitoring. Therefore, we did not run the analyses separately on a region-by-region manner. In addition, we needed to ensure enough voxels to be compared between conditions in calculation of similarity level. For example, it is possible that some regions have more voxels, and if we selected voxels depending on previously defined anatomical boundaries, it is difficult to rule out the alternative explanation of possible statistical insignificance of a specific region, such that it is not clear whether a null result was derived from either low reliability due to small numbers of voxels in the tested region or real null effects of a given region. Finally, we wanted to avoid subjective regional boundary criteria for making ROI masks separately given that voxels spread out widely across different regions (Figure 2A). Although Table 1 was prepared to give a general idea of what regions are involved in the anticipation process, it does not suggest clear boundaries of specific regions since the table indicates structural regions based on the peak coordinates that fell into more than 50% anatomical probability (i.e., Harvard-Oxford atlas) with more than 20 activated voxels. Thus, instead of investigating each region separately, we used all voxels activated in the cluster given that those regions were recruited as a network during the anticipation process.

#### Representational Similarity Analysis

We conducted the representational similarity analysis comparing neural patterns across the two runs (‘Observation’ vs. ‘Driving’). We decided to estimate the anticipatory pattern and outcome patterns from these two independent runs separately because we wanted (1) to see how mothers anticipate their child’s behaviors based on their own experiences as a consequence of their choices, and, (2) most importantly, to avoid signal overlaps between the anticipation phase and outcome onsets in the observation run since outcome events come right after the anticipation phase. For the estimation of anticipatory neural patterns, the General-Linear Model (GLM) analysis was performed for the ‘Observation’ run with the time period that starts 3 s before and ends 2 s after the yellow light (i.e., the time before they know their child’s decisions; Figure 1D). In addition, outcome onsets with a 1 s duration, temporal derivate regressors and motion regressors (six motion parameters and motion-outlier points) were included to the design matrix as nuisance regressors. For the ‘Driving’ run, the neural patterns for the outcomes (‘Safe’ or ‘Risky’) were modeled as 2 s after the yellow light offset. Unlike the observation run, mothers made decisions (‘Go’ and ‘Stop’) for each intersection, and we thus included all decision onsets and their durations as nuisance regressors with temporal derivate regressors and motion regressors (six motion parameters and motion-outlier points). One mother did not make any ‘Stop’ decisions, which yielded no ‘Safe’ outcomes, three mothers did not make any ‘Go’ decisions, which resulted in no ‘Risky’ outcomes. We excluded those mothers from the final analysis for the corresponding outcome. Because Crashes were rare (only 8 total possible, and most with 0–2 total crashes) ‘Crash’ outcomes were not analyzed, and we only focus on the ‘Safe’ and ‘Risky’ outcomes in the pattern similarity analysis. All the neural patterns were z-transformed estimates of the condition of interest based on the ‘Condition > baseline’ contrast (e.g., Safe outcome > Baseline).

To calculate the neural pattern similarity between mothers’ anticipation of adolescent risk taking and their own outcomes, we extracted neural patterns within the ROI (see the ROI selection, above) from ‘Anticipation’ during the ‘Observation’ run and from the outcomes (‘Safe’ and ‘Risky’) from the ‘Driving’ run, vectorized them, and computed the similarity metrics based on the cosine similarity estimation (Figure 2B). The cosine similarity is the cosine of the angle formed between two vectors, and the patterns are considered to be either identical if the cosine similarity equals 1 or dissimilar if the value equals −1 (Mitchell et al., 2008).

## Results

### Behavioral Results

On average, mothers made safe decisions (i.e., ‘Stop’) on 57.26% of trials (*M*_*number of decision*_ = 14.870, SD = 7.509, range: 0–26)^4^. Adolescents made safe decisions on 46.70% of trials (*M*_*number of decision*_ = 12.130, *SD* = 4.920, range: 2–21), though it was not significantly different from their mothers, *paired-t* (22) = 1.586, *p* = 0.127. Correlation analyses were conducted to examine the relationship between mothers’ safe decisions and their child’s safe decisions. Mothers’ percentage of safe decisions was not related to their children’s percentage of safe decisions, *r*(23) = 0.158, *p* = 0.417. Moreover, mothers’ parental monitoring was not associated with their own safe decisions, r(23) = −0.057, *p* = 0.795, their children’s safe decisions, *r*(23) = 0.163, *p* = 0.458, or their children’s self-reported risk-taking behavior, *r*(23) = −0.088, *p* = 0.689.

### Link Between Parental Monitoring and Neural Pattern Similarity

**FIGURE 3:**
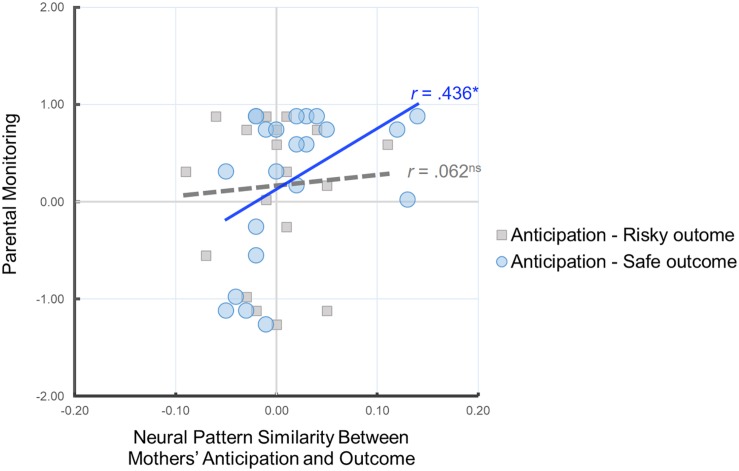
Scatter plot illustrating the relationship between mothers’ Anticipation-Safe Outcome/Anticipation-Risky Outcome pattern similarity within the anticipation network ROI mask and parental monitoring. Parental monitoring was mean-centered. ^∗^*p* < 0.05.

Our primary analysis examined whether the neural pattern similarity between mothers’ anticipation of child’s behavior and mothers’ own decisions is associated with parental monitoring. To this end, we conducted correlation analyses between the similarity value of the neural patterns and parental monitoring. As shown in Figure 3, we found that there was a significant positive correlation between parental monitoring and Anticipation-Safe Outcome similarity, *r*(22) = 0.436, *p* = 0.043, whereas there was no such association for Anticipation-Risky Outcome similarity, *r*(20) = 0.062, *p* = 0.796. In other words, mothers with higher parental monitoring show greater neural similarity between their neural representation of anticipating their child’s decisions and their own safe outcomes. Follow-up analysis comparing the two correlation coefficients for dependent samples indicated that the association between parental monitoring and Anticipation-Safe Outcome similarity is marginally larger than the association between parental monitoring and Anticipation-Risky Outcome similarity, *z* = 1.34, *p* = 0.09.

### Neural Representation of Parental Monitoring and Adolescent Risk-Taking Propensity

**FIGURE 4:**
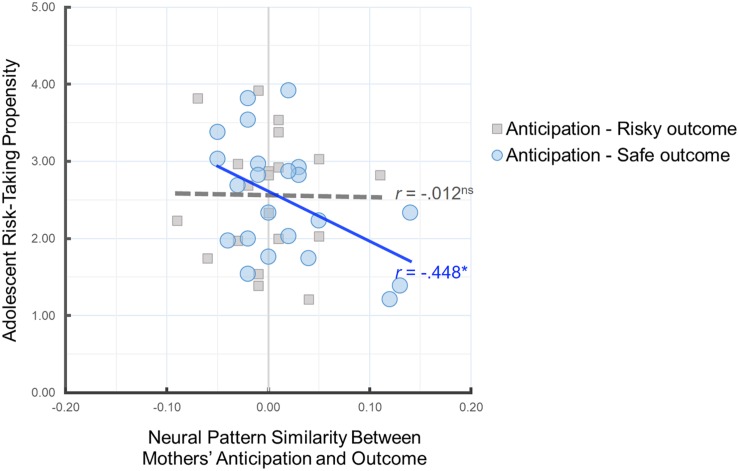
Scatter plot illustrating the relationship between mothers’ Anticipation-Safe Outcome/Anticipation-Risky Outcome pattern similarity within the anticipation network ROI mask and adolescent risk-taking propensity. ^∗^*p* < 0.05.

Next, we investigated the implications of parents’ neural pattern similarity for adolescents’ risk-taking propensity. To this end, we examined whether mothers’ neural pattern similarity during anticipation of their child’s decisions and their own outcomes is related to adolescent-reported risk-taking propensity. As shown in Figure 4, greater neural pattern similarity between mothers’ anticipation and their own safe outcomes was associated with lower risk-taking propensity in adolescents, *r*(22) = −0.448, *p* = 0.037, suggesting that the neural pattern similarity between mothers’ anticipation of their adolescent’s decision and their own safe outcomes serves as a meaningful index of parental monitoring and relates to adolescents’ risk-taking propensity. There was no association between Anticipation-Risky Outcome neural pattern similarity and adolescent risk taking, *r*(20) = −0.012, *p* = 0.960. Follow-up analysis comparing the two correlation coefficients for dependent samples indicated that the association between mothers’ Anticipation-Safe Outcome similarity and adolescent risk-taking propensity is marginally larger than the association between mothers’ Anticipation-Risky Outcome similarity and adolescent risk-taking propensity, z = 1.55, *p* = 0.06.

**FIGURE 5:**
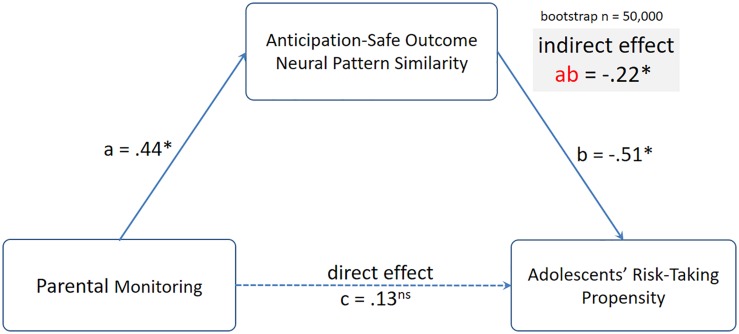
The indirect effect from parental monitoring to mothers’ Anticipation-Safe Outcome neural pattern similarity to adolescents’ risk-taking propensity was significant. Standardized coefficients from the mediation analyses are presented.

To further investigate these relationships, we conducted mediation analyses using the mediation toolbox^5^. In particular, we tested the hypothesis that the link between parental monitoring and adolescents’ risk-taking propensity was mediated by parents’ neural pattern similarity between mothers’ anticipation and their own safe outcomes. Despite no direct association between parental monitoring and adolescents’ risk-taking propensity, we examined the indirect effect from parental monitoring to Anticipation-Safe Outcome neural pattern similarity to adolescent risk-taking propensity. Using 50,000 resampling, the indirect effect from parental monitoring to Anticipation-Safe Outcome neural pattern similarity to adolescents’ risk-taking propensity was significant, indirect effect β = −0.22, 95% CI: [−0.52, −0.03] (see Figure 5).

## Discussion

As children enter the adolescent years, they are socially and biologically driven to seek individuation and exploration, with heightened risk-taking behavior, such as drinking, smoking, and substance use (Arnett, 1992; Casey et al., 2008; Steinberg, 2008). Although adolescents spend increasing time with their peers (Larson and Verma, 1999), parents still serve as important socialization agents (Collins and Steinberg, 2006; Smetana et al., 2006). Decades of research has consistently demonstrated the protective role of parental monitoring – parents’ effort of knowing adolescents’ activities and behavior – in preventing risk taking (e.g., Steinberg et al., 1994; Jacobson and Crockett, 2000; Li et al., 2000b; DiClemente et al., 2001; Mann et al., 2015). Despite accumulating neuroimaging studies on adolescent risk taking in the past decade, no prior study focuses on parents’ brain to shed light on the neural representation of parental monitoring. Using a representational similarity approach, the present study sought to identify neural representation of parental monitoring, with attention to implications for adolescent risk taking. Parental monitoring is reflected in the similarity between neural patterns of mothers’ anticipation of their adolescent child’s risk taking and experiencing their own safe outcomes, such that mothers with higher parental monitoring showed greater neural pattern similarity when anticipating their child’s decisions and their own safe outcomes. Moreover, such neural pattern similarity was associated with lower adolescent risk-taking propensity.

### Neural Pattern Similarity as Neural Representation of Parental Monitoring

Despite wide interest in the role of parents in adolescent adjustment, neuroimaging research on parents’ beliefs and practices is relatively scarce. Most studies in this line of research examine how parents’ beliefs and practices play a role in adolescents’ brain structure and function, with implications for adolescent adjustment (e.g., Olsavsky et al., 2013; Gee et al., 2014; Whittle et al., 2014, 2016; Qu et al., 2015, 2016; McCormick et al., 2016). To date, few studies focus on parents’ brain to explore the neural mechanisms of parents’ beliefs and practices (Noll et al., 2018). While prior neuroimaging research on parents’ brain has used a univariate approach (e.g., Musser et al., 2012; Piallini et al., 2015; Bornstein et al., 2017), the present study took a representational similarity approach to explore the neural representation of parental monitoring. To better capture parental monitoring, which involves parents’ effort to anticipate and know their adolescent’s behavior, we used a novel non-verbal design that can assess neural processes during parents’ anticipation of adolescent risk taking (i.e., in the observation run) as well as their own experience of making decisions and receiving outcomes (i.e., in the driving run). Indeed, we found that the anticipatory neural patterns of mothers with higher parental monitoring were more similar to the neural patterns when mothers received safe outcomes but not risky outcomes. These findings provide new insights into parental monitoring, suggesting that mothers who monitor their child’s activities may expect their child to make safe choices in a risk-taking context. Therefore, parental monitoring is more than just trying to know how adolescents would behave, but may also involve expecting their adolescents to engage in safe behavior, as reflected in the neural pattern similarity. Such implicit aspects of parental monitoring may provide additional insight into why parental monitoring plays a positive role in promoting children’s optimal adjustment.

### Implications of Neural Representation of Parental Monitoring for Adolescent Risk Taking

Given the protective role of parental monitoring (Dishion and McMahon, 1998; Stattin and Kerr, 2000; Fletcher et al., 2004), it is expected that neural representation of parental monitoring would be associated with adolescent risk taking. Indeed, we found that parental monitoring was linked to neural pattern similarity between mothers’ anticipation of their child’s decisions and experience of their own safe outcomes but not risky outcomes, and such neural pattern similarity was linked to lower risk-taking propensity in adolescents. These findings indicate that when parents anticipate their child’s decisions in a risky context, and when such anticipation is reflected by neural pattern similarity with their own safe behavior, their child tends to benefit and show lower risk-taking propensity. Although there was no significant link between parental monitoring and adolescent risk taking in the current study, such a direct effect is not necessary, in part because of the timing of the process, particularly in developmental research (e.g., MacKinnon et al., 2000; Shrout and Bolger, 2002; Hayes, 2009; Zhao et al., 2010; Rucker et al., 2011). In the current study, parental monitoring may ultimately have an effect on adolescent risk taking, but the effect may not be detectible as it takes time for parental monitoring to play a role. Taken together, these findings suggest that the similarity between neural representation of mothers’ anticipation and their safe outcomes is a meaningful index of parental monitoring and may be protective against their adolescents’ risk taking. It is possible that parents who monitor their child more socialize their children to make more safe decisions in risky contexts, leading their children to engage in less problem behavior in daily life. Therefore, our findings not only identify the neural representation of parental monitoring, but also highlight the importance of incorporating parents’ brain into consideration when examining adolescent adjustment.

### Limitations

Several limitations and future directions should be acknowledged. First, although the current study provides preliminary evidence on the neural representation of parental monitoring and its association with adolescent risk taking, the sample size is relatively small. Given the exploratory nature of this study, future research is needed to replicate the findings with a larger sample. Second, the current study only examines the concurrent association between neural representation of parental monitoring and adolescent risk taking, showing that greater neural pattern similarity between parents’ anticipation of adolescent risk taking and their own safe outcomes is related to lower likelihood of adolescent risk taking. However, it will be useful for future research to employ a longitudinal design and explore the role of neural representation of parental monitoring in longitudinal changes of adolescent risk taking over time, which is particularly important for testing mediational paths. The predictive power of such neural representation can provide more robust evidence and highlights the importance of incorporating parents’ brain into understanding of trajectories of adolescent behavior. Moreover, previous longitudinal evidence suggests that adolescents’ delinquency and drug use are predictive of lower parental monitoring over time, which is related to later delinquency and drug use (Aseltine, 1995; Barber, 1996). It will be interesting to examine the reciprocal relationships between neural representation of parental monitoring and adolescent risk taking over time. It is important to acknowledge that the current study, along with other empirical research on parental monitoring, relies on self-reported measure to assess parental monitoring and adolescent risk taking. Self-report approaches may suffer from social desirability. Past research suggests that self-report or child-report measures of parental monitoring still capture meaningful differences in parenting practices and are linked with lower risk-taking behavior among children and adolescents (e.g., Li et al., 2000a; Fletcher et al., 2004; Fosco et al., 2012). Although we did not collect data on child-reported parental monitoring in the current study, future research that employs a multi-faceted, multi-informant approach to assess parental monitoring is needed to examine the robustness of the current findings. Finally, it remains unclear why neural representation of parental monitoring is related to adolescent risk taking (i.e., the mechanisms through which neural representation of parental monitoring plays a role in adolescent risk taking). It is possible that adolescents’ neural responses serve as a mediator. Therefore, future studies can examine whether adolescents’ neural representation of risky and safe behavior explains the association between parents’ neural representation of parental monitoring and adolescent real-life risk taking.

## Conclusion

In conclusion, the current study examines the neural representation of parental monitoring. Using a novel paradigm that includes both parents’ anticipation of adolescent risk taking and their own risk taking, we demonstrated that parental monitoring is reflected in the similarity between neural pattern of anticipating their adolescents’ risk taking and experiencing of their own safe outcomes. Importantly, such neural pattern similarity is associated with less likelihood of adolescent risk taking. Taken together, such a neural pattern similarity approach can serve as an index of parental monitoring, with meaningful implications for adolescent risk taking.

## Data Availability Statement

The datasets generated for this study are available on request to the corresponding author.

## Ethics Statement

The studies involving human participants were reviewed and approved by Institutional Review Boards of the University of Illinois. Written informed consent to participate in this study was provided by the participants’ legal guardian/next of kin.

## Author Contributions

YQ and ET designed the research. YQ performed the research. T-HL and YQ analyzed the data. T-HL, YQ, and ET wrote the manuscript.

## Conflict of Interest

The authors declare that the research was conducted in the absence of any commercial or financial relationships that could be construed as a potential conflict of interest.

## Footnotes

1 The main results of adolescents’ Stoplight task have been reported elsewhere (Telzer et al., 2015; Guassi Moreira and Telzer, 2018).

2 http://screencast-o-matic.com

3 https://neurovault.org/images/132921

4 Risky decisions are the opposite of safe decisions, and thus the results are identical when examining risk decisions. We therefore only present these findings for safe decisions.

5 https://github.com/canlab/MediationToolbox
